# Development of a MD-LC-MS/MS Method to Analyze 3 Bioactive Compounds in Huoxuezhitong Rubber Patch and Application to a Pharmacokinetic Study in Rats

**DOI:** 10.1155/2019/6173565

**Published:** 2019-01-17

**Authors:** Yahua Cui, Chang Yang, Jun Liang, Linying Zhong, Caifeng Liu, Jie Bai, Shouying Du, Daofang Liu

**Affiliations:** ^1^School of Chinese Materia Medica, Beijing University of Chinese Medicine, Fangshan District 102488, Beijing, China; ^2^An Hui An Ke Yu Liang Qing Pharmaceutical Co., Ltd, Anqing 246001, Anhui, China

## Abstract

Huoxuezhitong rubber patch, a well-known traditional Chinese medicine (TCM) prescription, is utilized to treat pain and inflammation. In this study, a microdialysis-ultra-high-performance liquid chromatography-tandem mass spectrometry (MD-LC-MS/MS) method was designed for the simultaneous determination of active constituents in the rubber patch, such as paeonol (Pae), eugenol (Eug), and piperine (Pip). A microdialysis probe was implanted in the subcutaneous tissue of a rat, which is intended to detect the subcutaneous concentrations of target components. Saline containing 30% ethanol acted as perfusion fluid. Analytes in the microdialysate were completely separated over an ACQITY UPLC RBEH C_18_ column (2.1mm×100mm, 1.7*μ*m). The mobile phase was composed of 0.01% ammonia aqueous and acetonitrile-0.01% ammonia with gradient elution. The single-run analysis time was 10.0 minutes. The linear regression displayed good linear relationships in the ranges of 0.25–100 ng/mL for paeonol and eugenol and 0.001–5 ng/mL for piperine. The interday and intraday precision of the quality control samples exhibited relative standard deviations (RSD) <13.56%. The accuracy values ranged from −14.92% to 14.00%. The present method was successfully applied in pharmacokinetics studies following dermal administration of Huoxuezhitong rubber patch in rats. Pip's T_max_ (488.00±150.73) min was greater than that of Pae (186.67±48.44) min and Eug (240.00±138.56) min, and the rank order of t_1/2_ was Pae > Pip > Eug. The rank order of AUC_0-720_ and C_max_ was both Eug > Pae > Pip. MRT_0-∞_ of Pip was higher than that of Pae and Eug. Eugenol showed a faster elimination and a shorter half-life. Paeonol showed a stronger drug reservoir function after removing the drug source.

## 1. Introduction

Huoxuezhitong rubber patch is one of the well-known Chinese patent medicines employed to treat painful and inflammatory diseases such as knee osteoarthritis, knee joint synovitis, and lumbar disc herniation [1]. There are more than twenty herbs in this prescription. Moutan Cortex (MC) is the most important one for its anti-inflammatory, antispasmodic, and analgesic effects [2]. Clove, warm in nature and spicy in flavor, is effective in warming middle energizer, dispersing cold and relieving pain according to the theory of TCM. Eugenol contained in clove is thought to exert an analgesic effect [3]. Paeonol (Pae) from MC and eugenol (Eug) from clove are two main active components in Huoxuezhitong rubber patch. Pharmacological studies have demonstrated that paeonol has anti-inflammatory and analgesic effects [4–7], hepatoprotective [8], cardiovascular protection [9], and neuroprotective properties [10]. Eugenol is widely used in pharmaceutical production due to its anti-inflammatory effect [11–13], analgesic effect [14], antibacterial effect [15], antifungal effect [16], antioxidant [17], and anticancer activities [18]. Piperine (Pip), the major organic alkaloid compound rich in pepper, is good for treating various inflammatory diseases [19], such as gut inflammation [20]. In Chinese market, the total sales of Huoxuezhitong rubber patch are more than 10 billion RMB each year. Though the prescription is used widely, its rubber matrix with low water content may cause slow and brief drug release, especially compared with the gel matrix [21].

To our knowledge, the local pharmacokinetic properties of formula loading pure ingredients have been investigated [22, 23], while there are few studies about the compound prescription, especially following dermal administration. Thus, the study of pharmacokinetic parameters of a standard compound will be helpful to evaluate the efficacy and safety in clinical application.

Huoxuezhitong rubber patch is an external preparation. As an interface between the organism and the external environment, stratum corneum (SC), the first defensive line of human body plays a major role in protecting and supporting its surrounding life [24]. Large storage capacity and low transport capacity are the most noticeable characteristics of SC. For external preparation, drug must penetrate the SC barrier to obtain its clinical value; the subcutaneous drug level directly affects the therapeutic effect. Thus, it is necessary to obtain the subcutaneous drug concentration to ensure the efficacy of the traditional medicine. However, compared with oral or inject administration, the sampling of bioactive compound in subcutaneous tissue is much more difficult [25–27]. Thanks to the new technique—Dermal Microdialysis, we can obtain enough samples for the subsequent analysis.

Microdialysis technology (MD), by monitoring the biochemical milieu around the probe-membrane [28, 29], provides a great convenience for the study of local pharmacokinetics [30]. Probe-membrane is a key component in a Microdialysis system. Due to constant flow of perfusate inside the probe-membrane, absolute equilibration is not usually attained between the perfusate and extracellular fluid (ECF) of the surrounding tissue. This implies that only a part of the actual drug is collected in the dialysate. Therefore it is a prerequisite to correct the probe recovery for the application of microdialysis technology. In addition, for nonpolar compounds, it has been found that the real concentration of the drug in the outlet is lower than the theoretical one [31]. This suggests the microdialysis probe may lead to slight adsorption of liposoluble components [3], such as Pae, Eug, and Pip, and then result in the variety of probe recovery. Hence before the animal experiment, the perfusion fluid must be selected first to reduce the adsorption.

In this study, we established a direct and effective MD-LC-MS/MS method to simultaneously quantify three major ingredients of Huoxuezhitong prescription. The validated method was successfully applied to local pharmacokinetic study of Huoxuezhitong rubber patch following dermal administration.

## 2. Materials and Methods

### 2.1. Chemicals and Reagents

The standard Pae and Pip were purchased from Chengdu Mansi Biotechnology Co., Ltd. Chengdu Institute of Biology, Chinese Academy of Sciences (Chengdu, China). The standard Eug was available from the National Institute for Control of Pharmaceutical and Biological Products (Beijing, China). HPLC-grade acetonitrile was provided by Merck (USA). Huoxuezhitong Gao was provided by An Hui An Ke Yu Liang Qing Pharmaceutical Co., Ltd. Microdialysis probes (CMA 30 Linear MD Probe 4/pkg) used in this study were purchased from Sweden US Office (material: polyimide; membrane length: 10mm; cut-off: 6000 Dalton).

### 2.2. Animals

All male Sprague-Dawley (SD) rats (weighing 200 ± 10g) were purchased from Beijing Vital River Laboratory Animal Technology Co., Ltd. (Beijing, China). All animals were housed in the Beijing University of Chinese Medicine Laboratory. Animals were kept in a controlled environment with consistent temperature and humidity, 12-h light/12-h dark cycle, and abundant food and water. All the animal studies were performed under the Guidelines for the Care and Use of Laboratory animals and the experimental protocols were approved by the institutional animal experimentation committee of Beijing University of Chinese Medicine.

### 2.3. Microdialysis

#### 2.3.1. The Selection of Perfusion Fluid

Pae, Eug, and Pip are both liposoluble components. To alleviate the adsorption to the probe membrane, different kinds of organic solution were added to the perfusion fluid. The four kinds of perfusion fluid were saline containing 20% propylene glycol (20%PG-NS), saline containing 10% propylene glycol and 10% ethanol (10%PG-10%Et-NS), saline containing 20% ethanol (20% Et-NS), and saline containing 30% ethanol (30% Et-NS).

The linear microdialysis probe was immersed in stirred (350 r·min^−1^) and temperature-controlled (32.5°C) perfusion fluid. One end of the microdialysis probe was connected to microsyringe, loading analyte-free perfusion fluid. On the other end, samples were collected in 200 *μ*L glass tubes. Before collecting, the microdialysis system was perfused with blank perfusion fluid at a flow rate of 1.5 *μ*L/min for 1 hour with a Pump 11 Elite (Harvard Apparatus). Then the microdialysis system was perfused with perfusion fluid containing a known amount of Pae. Microdialysis samples were collected every 20 minutes. The sample collection lasted for 140 minutes. Then perfusion fluid containing Pae was replaced by pure perfusion fluid, simultaneously another eleven samples were taken. We determined the content of the drug in the samples by high performance liquid chromatography (HPLC), which we have reported in previous study [32]. The adsorption rate (AR) of different perfusion fluid was determined using a reverse dialysis method. AR was calculated as the formula (1). Then the adsorption rate-time curve for Pae was drawn.

(1) $$\begin{array}{c} AR\%=\frac{C_{t}}{C_{0}}\times 100 \end{array}$$

(C_t_: drug concentration at some point; C_0_: maximum concentration of all points)

#### 2.3.2. Surgical Procedures

Before surgery, a rat with the hair on the abdomen shaved off was anesthetized with Urethane (1.5g/ kg, intraperitoneal injection). Its shell temperature was maintained at about 32.5°C during the experiment with the help of an infrared lamp. The linear microdialysis probe was inserted through a 23-gage intravenous needle. Subsequently, the needle was taken out, leaving the dialysis membranes horizontal under the hairless skin.

#### 2.3.3. Microdialysis Probe Calibration

In our previous study, we determined the recovery rate (RR) of the three target components in vivo. Recovery rate for Pae, Eug, and Pip remains at (46.80±1.66)%, (45.06±1.66)%, and (34.15±2.06)%, respectively [32]. The RR would be used to calculate the real drug concentration in the subcutaneous tissue in pharmacokinetics studies.

### 2.4. LC–MS/MS

We have tried to determine the concentration of subcutaneous drugs using HPLC. However, the sensitivity of HPLC is not enough to quantify the amount of Pae, Eug, and Pip subcutaneous. So liquid chromatography was performed using a Nexera UHPLC LC-30A system (Kyoto, Japan) connected to a Sciex QTRAP 4500 tandem mass spectrometer (Applied Biosystems). Data acquisitions were made utilizing SCIEX Analyst software (v.1.6). Compounds were separated over an ACQUITY UPLC RBEH C_18_ column (2.1mm×100mm, 1.7*μ*m). When detecting Pae and Eug, mobile phases consisted of water containing 0.01% ammonia (mobile phase A) and acetonitrile (ACN) containing 0.01% ammonia (mobile phase B). Gradient employed went from initial 30:70 (A: B) to 50:50 (A: B) in 2 min, held for 3 min. Then the gradient went from 50:50(A: B) at 5 min up to 100:0 (A: B) at 6 min, retained for 1 min. The gradient went back to 30:70 (A: B) in 0.1 min. This was maintained until the end of the analysis. When detecting Pip, mobile phases consisted of water containing 0.1% formic acid (mobile phase A) and acetonitrile-methanol (2:8, v: v) mixture containing 0.1% formic acid (mobile phase B). Gradient employed went from initial 30:70 (A: B) to 50:50 (A: B) in 2 min, held for 1 min. Then the gradient went from 50:50(A: B) at 3 min up to 100:0 (A: B) at 4 min, retained for 1 min. The gradient went back to 30:70 (A: B) in 0.1 min. This was maintained until the end of the analysis. The chromatographic run time for each sample was 10 minutes at a flow rate of 0.2 mL/min. The injection volume was 10 *μ*L and the column temperature was set at 25°C and the autosampler temperature at 4°C.

Mass spectrometry detection was carried out in the negative ion mode for Pae and Eug, positive ion mode for Pip using an electrospray ionization (ESI) source. The optimised MS parameters were conducted using multiple reaction monitoring, as described in Table 1. The operation conditions of Pae and Eug were as follows: ion spray voltage, -4500 V; curtain gas, 10 psi; ion source gas 1, gas 2 both at 40 psi; source temperature at 200°C. The following were the operation conditions of Pip: ion spray voltage, 3500 V; curtain gas, 10 psi; ion source gas 1, gas 2 both at 50 psi; source temperature at 500°C.

### 2.5. Blank Microdialysate Preparation

The microdialysis probe was implanted in accordance with the procedure described in Section 2.3.2. The microdialysis system was perfused with 30% ethanol solution at the flow rate of 1.5 *μ*L/min to collect enough blank microdialysates. Then, the samples were immediately stored at −80°C until detected. If the rat suddenly died, which happened almost rarely, the collection had to be terminated.

### 2.6. Standard Solutions Preparation

Reference standards were accurately weighed and dissolved in methanol to prepare mixed stock solutions, with concentrations of 1mg/mL for target compositions. Solutions of 10 *μ*g/mL Pae, Eug, and Pip were obtained by diluting mixed stock solutions with a mixture of methanol: water (30: 70, v/v). They were serially diluted with blank microdialysates to obtain calibration standard stock solutions of gradient concentrations.

### 2.7. Method Validation

#### 2.7.1. Selectivity

The selectivity was demonstrated by comparing the chromatograms of blank microdialysates, blank microdialysates spiked with analytes, and microdialysis samples.

#### 2.7.2. Matrix Effect

The matrix effects were investigated by comparing the peak areas acquired from blank microdialysates spiked with standard solutions with the pure standard solutions at three different concentrations (n=6). Analyte matrix effects were calculated from peak area ratios as formula (2):

(2) Matrix  effect%=Analyte  peak  area  in  free  microdialysatesAnalyte  peak  area  in  pure  solutions×100

#### 2.7.3. Linearity and Lower Limits of Quantification (LLOQ)

Linearity was assessed by plotting the peak area of the analyte versus the concentration of the calibration standards (six calibration points from blank samples spiked with the standards: 0.25, 0.5, 1, 2.5, 5, 10, 25, 50, and100.0 ng/mL for Pae and Eug, 0.001, 0.005, 0.25, 0.1, 0.25, 0.5, 1, 2.5, and 5 ng/mL for Pip). Calibration curves were described in the form of y = a + bx (1/x^2^ weighted), where y is the peak area of the analytes and x (ng/mL) is the concentration of the calibration standards. Calibration curves had to be constructed freshly for each experiment. The LLOQ was defined as the lowest concentration on the calibration curve with an acceptable accuracy error within ±20% and a precision less than 20%.

#### 2.7.4. Precision and Accuracy

The intra- and interday precision and accuracy were estimated by determining six replicate quality control samples (QC) at three concentration (low, medium and high) levels on the same day and three consecutive days, respectively. The relative standard deviation (RSD) acceptable for both of intra- and interday precision was required to be less than 15% and the acceptable relative error (RE) for the accuracy was required to be within 15% for all the QC samples.

#### 2.7.5. Stability

The stability of the analytes in blank microdialysates was investigated by analyzing the QC samples at three concentrations exposed to detection conditions. The prepared samples were set on a 4°C autosampler rack and were evaluated within 12 and 24 hours. When the accuracy is less than 15% of the rated concentration, the analyte is considered to be stable.

### 2.8. Pharmacokinetic Studies

Implantation and pretreatment of the probe are the same as that described in Section 2.3. Then a round rubber patch (3.46 cm^2^) was pasted on the hairless abdomen of the rat, respectively. After 720 min, the paste was scripted away. The skin was wiped softly and carefully to remove the residual drug. Simultaneously, another three samples was collected. If the rats suddenly died, the collection had to be terminated. All samples were immediately stored at 4°C until injected into the LC/MS/MS system for analysis. The sample concentration was corrected by RR.

The pharmacokinetics analysis was conducted by a noncompartmental approach using the Kinetica 5.1. The pharmacokinetics parameters, such as the maximum subcutaneous concentration (C_max_), time of reaching C_max_ (T_max_), the area subcutaneous concentration-time curve (AUC_0-t_), the mean residence time (MRT), and the terminal elimination half-life (t_1/2_), were calculated on each individual analyte.

## 3. Results and Discussion

### 3.1. Perfusion Fluid Selection

For water-soluble ingredients, the application of microdialysis technology following transdermal administration has become more mature [33, 34]. As for lipophilic ingredients, the application is greatly limited on account of the adsorption nature of the microdialysis probe. In order to alleviate the adsorption of fat-soluble components, we compared the AR of different perfusion fluid, using Pae as the test drug.

**Figure 1:**
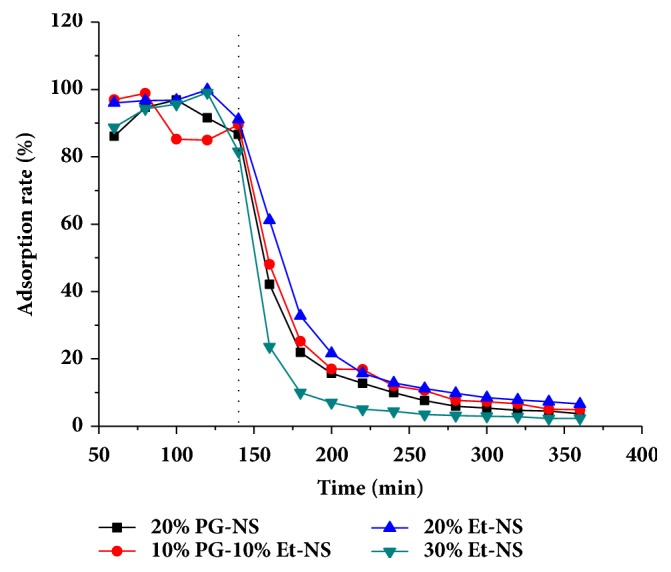
Adsorption rate-time curve for paeonol (reverse dialysis method) (n=2).

The results indicated that the linear microdialysis probe had different adsorption capacity to four kinds of perfusion fluid (Figure 1). The slope of different perfusion fluid indicated that 30% Et-NS had a sharp drop during 140 min to 200 min, while 20% Et-NS had a much slower one. From 200 min to 300 min, the AR for the four separate perfusion fluid was declining slightly and it began to keep stabilization after 300 min. At the last sampling point, the AR for 20%PG-NS, 10%PG-10%Et-NS, 20% Et-NS, and 30% Et-NS was, respectively, (3.72±0.35) %, (4.90±1.67) %, (6.58±1.13) %, and (2.37±1.32) %. The height of the plateau curve between 200 min and 360 min illustrated the order of the AR. As a result, 20% Et-NS lead to the maximum adsorption, while the linear microdialysis probe showed the minimum adsorption when it was perfused by 30% Et-NS. (The dotted line in Figure 1 indicated that perfusion fluid containing drug was replaced by blank perfusion fluid at that point.) So, 30% Et-NS was selected as perfusion fluid in this study. Perfusion fluid containing ethanol is suspected to cause the osmotic pressure difference between inside and outside the probe membrane. This leads to an effect on the stability of the recovery. However, a little amount of ethanol must be added to obtain accurate drug concentration in the subcutaneous tissue.

### 3.2. Method Validation

The composition of Huoxuezhitong prescription is complex, which increases the difficulty of separation and detection. To optimize the MS conditions, mobile phases mixed with acid and ammonia were used for the detection of the index component, respectively. Ionisation of the compounds was performed by electrospray ionization. The analytes were tested in both positive and negative ionization in the multiple reaction monitoring (MRM) modus. Stable quasimolecular ion peaks and perfect peak profile were obtained when using mobile phases mixing ammonia. Unlike normal pharmacokinetic study, we did not adopt an internal standard method to quantify the drug. For microdialysis, there are two methods to add internal standard: adding in the perfusion fluid, or adding in the final samples. But for this experiment, it may lead to a variable concentration of the internal standard during dialysis or lead to a dilution in the final sample. And since the analytical samples in this study were obtained through the membrane of microdialysis probe under the skin, with a cut-off of 6000 Dalton, there was no other processing procedure. Therefore, to obtain enough samples for the detection limit of MS, no internal standard was employed in this study.

#### 3.2.1. Selectivity

**Figure 2:**
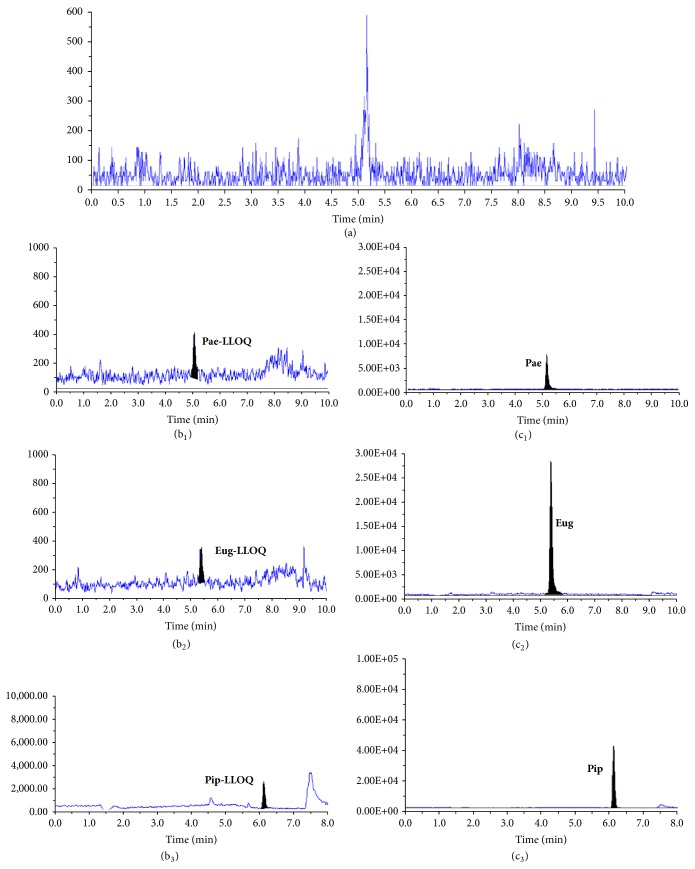
Representative MRM chromatograms of the analytes: (a) blank microdialysates; (b) blank microdialysates spiked with analytes at LLOQ; (c) sample collected at 10 h after a transdermal administration of Huoxuezhitong rubber patch in rats.

The typical chromatograms of blank microdialysates, blank microdialysates spiked with analytes, and microdialysis samples are shown in Figure 2. Under the established chromatographic conditions, there was no significant endogenous interference. No cross-interference was observed at the retention time of the analytes.

#### 3.2.2. Matrix Effect

The matrix effects of all QC samples were observed in the range from 86.14% to 114.79%. It was within the acceptable range. All of the RSD values were less than 15.00%. Obviously, endogenous substances produced no significant effect on the transdermal samples. The results were shown in Table 2.

#### 3.2.3. Linearity and Sensitivity

Only one standard curve was prepared because the drug under the skin had little concentration fluctuations at the same sampling point for transdermal formulation. The calibration curve was y = 7759.3x – 904.92, with r value of 0.9997 for Pae; y = 6207.2x – 2248.1, with r value of 0.9997 for Eug; y = 4000000x+256472, with r value of 0.9943 for Pip. The linear regression displayed good linear relationships over the ranges of 0.25–100 ng/mL for Pae and Eug. The lower limit of quantification (LLOQ) was 0.25 ng/mL for both. The linear regression displayed good linear relationships over the ranges of 0.001–5 ng/mL for Pip, with LLOQ 0.001 ng/mL.

#### 3.2.4. Precision and Accuracy

Results summarized in Table 3 were the intraday and interday precision and accuracy for the three compounds from the QC samples at three concentration levels. The precision and accuracy conformed to the acceptable range for biological media.

#### 3.2.5. Stability

The results displayed in Table 4 indicated that the target compounds were stable in blank microdialysate for 12 and 24 hours, with RSD values less than 20% for low concentration and 15% for medium and high concentration.

### 3.3. Pharmacokinetic Studies

**Figure 3:**
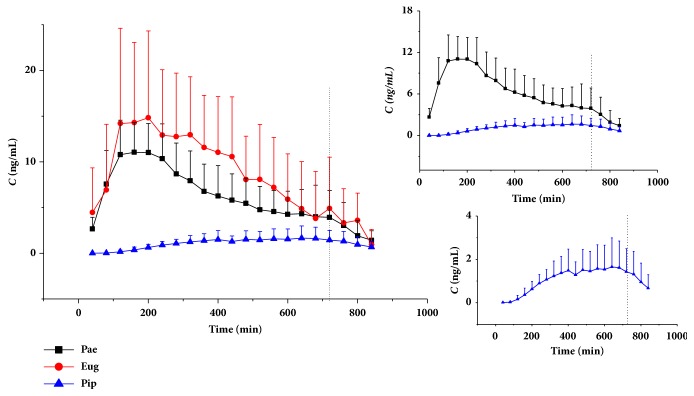
The profiles of mean transdermal concentration-time of paeonol, eugenol and piperine after transdermal administration of Huoxuezhitong rubber patch to rat (mean ± SD, n = 6).

The established LC–MS/MS method was successfully applied to the pharmacokinetic study of Huoxuezhitong rubber patch. Mean transdermal concentration-time profiles of the three analytes were presented in Figure 3 (The dotted line in Figure 3 indicated the rubber patch was scripted at that point.) All of the pharmacokinetics parameters calculated by the noncompartment model were summarized in Table 5. It was found that Pip's T_max_ (488.00±150.73) min was greater than that of Pae (186.67±48.44) min and Eug (240.00±138.56) min and the rank order of t_1/2_ was Pae > Pip > Eug. This revealed that Pae eliminated more slowly. The rank order of AUC_0-720_ and C_max_ was both Eug > Pae > Pip. MRT_0-∞_ of Pip was higher than that of Pae and Eug. This indicated that the drug storage cavern of Pip under the skin was bigger. As for Pae and Eug, lower MRT_0-720_ and higher MRT_0-∞_ implied a strong reservoir function for Pae in the skin.

The structure and physical parameters of the three analytes are shown in Table 6. Pae and Eug have small molecular weight and low melting points. These makes ingredients penetrate into skin easily [35–37], while Pip has a larger molecular weight and high melting points, which can help explain the results of slow penetration. Because of its smaller polarity, eugenol has a lower tissue affinity, which may be a reason why eugenol showed a faster elimination and a shorter half-life. Before the rubber patch was removed, the cumulative amounts of subcutaneous of both components were increasing. Paeonol showed a stronger drug reservoir function after removing the drug source.

## 4. Conclusions

Huoxuezhitong rubber patch consists of 28 herbs and extracts. The complexity greatly increases the difficulty in analysis. In this study, we chose three main anti-inflammatory compounds-paeonol, eugenol, and piperine as index. An accurate and rapid MD-LC–MS/MS analytical method was established and validated for the simultaneous determination of the three liposoluble compounds in rats' subcutaneous tissue for the first time. The MD-LC–MS/MS method was successfully applied to the pharmacokinetics study of Huoxuezhitong rubber patch. The results might offer some references for the clinical use of this formula.

## Figures and Tables

**Table 1:** List of the analytes and the corresponding MRM parameters. *Abbreviations.* MRM: multiple reactions monitoring; DP: declustering potential (V); EP: entrance potential (V); CE: collision energy (V); CXP: cell exit potential (V).

Substance	MRM_1_	MRM_2_	DP	EP	CE	CXP
Pae	165	122/150	-85	-7	-30/-22	-30/-35
Eug	163	148	-50	-10	-18	-10
Pip	286.2	200.9/135	70	5	27/33	15/10

**Table 2:** The matrix effects of QC samples at three concentrations (n=6).

Concentration (ng/mL)	Matrix effect	
	Mean ± SD(ng/mL)	RSD (%)
Paeonol		
1	101.65±10.36	10.19
20	90.31±3.03	3.35
80	96.81±4.33	4.48
Eugenol		
1	97.59±13.23	13.55
20	108.63±10.23	9.42
80	109.56±5.60	5.11
Piperine		
0.003	93.35±0.88	0.94
0.1	99.21±0.72	0.73
4	100.2±0.17	0.17

**Table 3:** Accuracy and precision for the analytes in blank microdialysate (three validation days, six replicates at each concentration level per day) (n=6).

Concentration (ng/mL)	Precision, RSD (%)		Accuracy, RE (%)	
	Intra-day	Inter-day	Intra-day	Inter-day
Paeonol				
1	5.82	12.4	-11.82	-12.23
20	1.66	5.21	-14.92	-10.61
80	2.51	3.64	-9.65	-7.61
Eugenol				
1	4.93	11.76	6.07	-6.54
20	2.52	8.83	-12.42	-12.67
80	1.81	3.67	-5.27	-8.3
Piperine				
0.003	13.56	9.8	0.22	0.07
0.1	1.02	7.38	14.00	13.72
4	1.32	10.95	10.96	11.07

**Table 4:** Stability of the three analytes in blank microdialysate (n = 3).

Concentration (ng/mL)	Room temperature for 12 h	Room temperature for 24 h
	RSD (%)	RSD (%)
Paeonol		
1	4.12	12.16
20	2.09	4.1
80	0.28	4.05
Eugenol		
1	15.92	11.9
20	3.91	3.51
80	2.71	7.11
Piperine		
0.003	13.59	17.55
0.1	1.84	1.30
4	13.51	7.42

**Table 5:** Pharmacokinetic parameters of the 2 investigated compounds in male SD rats after transdermal administration Huoxuezhitong rubber patch (mean ± SD, n = 6).

Analytes	C_max_ (ng/mL)	T_max_ (min)	AUC_0-720_ (*μ*g/mL·min)	t_1/2_ (min)	MRT_0-720_ (min)	MRT_0-*∞*_ (min)
paeonol	12.59±3.29	186.67±48.44	5.02±1.94	336.98±228.04	363.37±53.40	570.07±316.75
eugenol	18.87±7.11	240.00±138.56	7.12±3.81	228.33±161.87	372.20±80.35	466.03±220.48
piperine	1.80±1.22	488.00±150.73	0.88±0.53	336.94±44.44	550.47±66.44	690.20±97.33

**Table 6:** Physical parameters of paeonol and eugenol.

	paeonol	eugenol	piperine
Structure	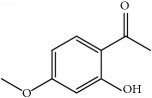	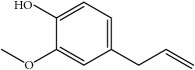	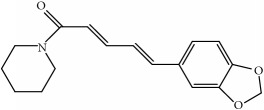
Molecular weight	166.18	164.20	285.34
melting point	48-50°C	−9.2°C-−9.1°C	131-135°C
Water solubility	349.8 *μ*g/mL	insoluble	insoluble

## Data Availability

The data used to support the findings of this study are available from the corresponding author upon request.
